# Decreased Bone Formation Explains Osteoporosis in a Genetic Mouse Model of Hemochromatosiss

**DOI:** 10.1371/journal.pone.0148292

**Published:** 2016-02-01

**Authors:** Mathilde Doyard, Daniel Chappard, Patricia Leroyer, Marie-Paule Roth, Olivier Loréal, Pascal Guggenbuhl

**Affiliations:** 1 INSERM UMR U991, F- 35033, Rennes, France; 2 Université de Rennes 1, IFR 140, F- 35043, Rennes, France; 3 GEROM Groupe Etude Remodelage Osseux et bioMatériaux LHEA, IRIS-IBM, Institut de biologie en santé, CHU, F- 49933, Angers, France; 4 INSERM U1043, F- 31300, Toulouse, France; 5 Service des Maladies du Foie, Hôpital Pontchaillou, CHU, F- 35033, Rennes, France; 6 Service de Rhumatologie, Hôpital Sud, CHU, F- 35033, Rennes, France; Lady Davis Institute for Medical Research/McGill University, CANADA

## Abstract

Osteoporosis may complicate iron overload diseases such as genetic hemochromatosis. However, molecular mechanisms involved in the iron-related osteoporosis remains poorly understood. Recent *in vitro* studies support a role of osteoblast impairment in iron-related osteoporosis. Our aim was to analyse the impact of excess iron in *Hfe*^*-/-*^ mice on osteoblast activity and on bone microarchitecture. We studied the bone formation rate, a dynamic parameter reflecting osteoblast activity, and the bone phenotype of *Hfe*^*−/−*^ male mice, a mouse model of human hemochromatosis, by using histomorphometry. *Hfe*^*−/−*^ animals were sacrificed at 6 months and compared to controls. We found that bone contains excess iron associated with increased hepatic iron concentration in *Hfe*^*−/−*^ mice. We have shown that animals with iron overload have decreased bone formation rate, suggesting a direct impact of iron excess on active osteoblasts number. For bone mass parameters, we showed that iron deposition was associated with bone loss by producing microarchitectural impairment with a decreased tendency in bone trabecular volume and trabecular number. A disorganization of trabecular network was found with marrow spaces increased, which was confirmed by enhanced trabecular separation and star volume of marrow spaces. These microarchitectural changes led to a loss of connectivity and complexity in the trabecular network, which was confirmed by decreased interconnectivity index and increased Minkowski’s fractal dimension. Our results suggest for the first time in a genetic hemochromatosis mouse model, that iron overload decreases bone formation and leads to alterations in bone mass and microarchitecture. These observations support a negative effect of iron on osteoblast recruitment and/or function, which may contribute to iron-related osteoporosis.

## Introduction

Genetic hemochromatosis (GH) related to the p.Cys282Tyr (C282Y) mutation in the HFE gene is one of the most prevalent genetic diseases worldwide. Genetic predisposition to the disease is homozygosity for the C282Y mutation, which is present in 3 out of every 1000 Caucasian persons with incomplete penetrance. GH leads to the development of progressive iron overload involving several tissues, including the liver, pancreas, or heart [1], resulting in life-threatening complications, such as cirrhosis, diabetes, and heart failure [2,3]. In addition, other clinical complications that worsen patients quality of life, including osteoporosis [4], have been reported. Osteoporosis is a bone disorder that increases fracture risk with low energy trauma and is defined by the World Health Organization as a decrease in bone mass and deterioration of bone microarchitecture [5]. As suggested by this definition, and demonstrated in many studies [6,7], alteration of the bone microarchitecture is an independent risk factor for fracture.

Thus, decreased bone mineral density has been reported in males presenting iron overload related to GH [8–10], similar to patients exhibiting secondary iron overload, such as thalassemia [11–14]. These data strongly support a deleterious impact of excess iron on bone structure, which exposes patients to fractures. Moreover, reports of increased iron in women after menopause support a potential additional impact of the iron parameters on the development of osteoporosis during the post-menopausal period [15]. The mechanisms involved in the development of iron overload-related osteoporosis are not fully understood. Whether altered bone remodelling is related to decreased osteoblast activity, reducing bone formation, and/or to increased osteoclast activity remains unclear.

Human histological studies of iron-overload impact on bones are old, few in numbers, and distorted by co-founding factor [16,17]. However, iron deposits have been found in genetic hemochromatosis patient’s bones. Regarding parameters bone remodeling, results are conflicting. Recent *in vitro* studies on rat foetal calvaria cultures and in osteoblast cell lines suggest a negative impact of iron on osteoblast functions [18–21], supporting a role of osteoblast impairment in iron-related osteoporosis. Moreover, iron has been shown to inhibit bone crystal growth via carbonate substitution [22]. An *in vivo* study of iron-overloaded pigs reported decreased osteoblast activity [23]. In rodents submitted to exogenous iron overload, the presence of iron deposits was associated with low bone mass and increased of bone remodelling [24] or loss of connectivity in trabecular bone [25]. More recently, in *Hfe*^*-/-*^ mice mimicking human GH, a relationship was found between iron overload and increased osteoclast number. In addition, both a low bone mass and disorganization of the bone microarchitecture was found in these mice [26].

Taken together, these elements suggest significant bone loss during iron overload, especially when related to GH or thalassemia. The diagnosis of hemochromatosis is currently made very early, and patients generally do not present with severe iron overload, visceral complications, or hypogonadism [27]. However, recent clinical studies have shown that the prevalence of osteoporosis has not decreased in *HFE*-hemochromatosis patients [8,9,10]. This finding suggests that even mild iron overload could impact bone metabolism.

Understanding the impact of iron excess on bone will be helpful for improving the follow-up of patients with excess iron, regardless of aetiology. Therefore, our aim was to analyse the impact of iron overload on bone quality *in vivo* in *Hfe*^-/-^ mice, a mouse model of human genetic hemochromatosis, with particular focus on osteoblast activity.

## Materials and Methods

### Animals

Male C57BL/6 *Hfe*^*-/-*^ mice (six months of age, n = 7) were used in this study. Male wild-type C57BL/6 (six months of age, n = 7) mice were used as controls. Animals were maintained at the Institut Fédératif de Recherche 140 animal facilities under standard conditions for temperature, atmosphere, and light. The animals had free access to tap water and SDS RM3(E) (Dietex, France) food. Each mouse received an intra-peritoneal injection of calcein (10 mg/kg body weight; Sigma 0875-10G) 7 and 2 days before euthanasia in order to perform a histodynamic analysis of the bone formation rate. Mice were anesthetized with Rompun® 2% [Xylazine]—Imalgène® 500 [Ketamine] solution and sacrificed by cervical dislocation at 6 months (*Hfe*^−/−^ males, H6M, n = 7; controls, C6M, n = 7). Tibia were dissected and fixed in 70% ethanol with 1% acetic acid for 24 h at 4°C, and then incubated in acetone. The liver was collected in order to determine the hepatic iron concentration (HIC).

Experimental procedures were performed in agreement with French laws and regulations (2010/63/UE). The protocol was approved by the Committee on the Ethics of Animal Experiments of Rennes (R-2010-OL-02). All efforts were made to minimize suffering.

### Iron concentration in the liver

The hepatic iron concentration (HIC) was determined according to Barry and Sherlock's biochemical method [28]. Results were expressed as micromoles of iron per gram of dry liver weight.

### Bone histomorphometry

Undecalcified tibias were embedded in methylmethacrylate at 4°C to maintain enzyme activity, particularly osteoclastic tartrate-resistant acid phosphatase (TRAcP). All histological techniques have been described elsewhere [29]. Sections (7 μm thick) were cut dry on a heavy-duty microtome equipped with 50° tungsten carbide knives (Leica Polycut S, Rueil-Malmaison, France). Parameters reflecting bone formation and resorption were measured on a semi-automatic image analyser system consisting of a Summasketch III digitizing tablet coupled to a PC and lab-made program. Measurements were taken in the secondary spongiosa, an area reflecting remodelling events similar to those in humans and located 1 mm under the growth cartilage at a magnification of ×200. The histomorphometric parameters were recorded in compliance with the recommendations of the American Society for Bone and Mineral Research (ASBMR) Histomorphometry Nomenclature Committee: trabecular bone volume (BV/TV, expressed as %), trabecular number (Tb.N, expressed in per mm), trabecular separation (Tb.Sp, expressed in μm), trabecular thickness (Tb.Th, expressed in μm), interconnectivity index (ICI), star volume of marrow spaces and trabeculae (V*_m.space_ and V*_trab_, respectively, expressed in mm^3^), and Minkowski’s fractal dimension (D_M_). For each mouse, four sections were stained with modified Goldner’s trichrome and used to measure osteoid parameters: relative osteoid volume (OV/BV, expressed as %) and osteoid thickness (O.Th, expressed in μm). The number of osteoclasts per bone area (N.Oc/B.Ar) were measured in TRAcP-stained sections. Perls' staining was performed on additional sections, and the fraction of the trabecular surface covered by an iron deposit (Fe-labelled surface) was estimated (Fe.LS/BS, expressed as %). Cancellous and cortical mineralization rates (Cn.MAR and Ct.MAR, respectively, expressed in μm/day) and double labelled surfaces (dLS/BS, expressed as %) were counted. Because of non-specific labelling of eroded surfaces, only the double labelled surfaces were taken into account. The bone formation rate (BFR/BS, expressed in mm^3^/mm^2^/day) was derived as Cn.MAR* dLS/BS*3.65.

### Statistical analysis

Statistical analyses were performed using SPSS 19.0 software. Data are expressed as mean ± SEM. The t-test was used to compare group means. Differences were considered significant when p≤0.05.

## Results

### General and iron parameters

No significant difference was found in final body weights between control and *Hfe*^*-/-*^ mice (Table 1). As expected, *Hfe*^*-/-*^ mice exhibited a significant increase in HIC compared to control mice.

**Table 1:** General and iron parameters in 6 months old control (C6M) and *Hfe*^*-/-*^ (H6M) male mice.

Mean ± SD	C6M (n = 7)	H6M (n = 7)	*p*
**Final Body Weight g**	32.71 ± 1.35	33.31 ± 2.37	NS
**HIC μmol iron/g liver**	***4*.*93 ± 1*.*59***	***18 ± 4*.*08***	***0*.*004***
**FeL.S/BS %**	***0***	***37*.*36 ± 23*.*64***	***0*.*006***

**HIC**: Hepatic Iron Concentration, **FeL.S/BS:** Fe-Labelled Surface/Bone Surface

**Fig 1:**
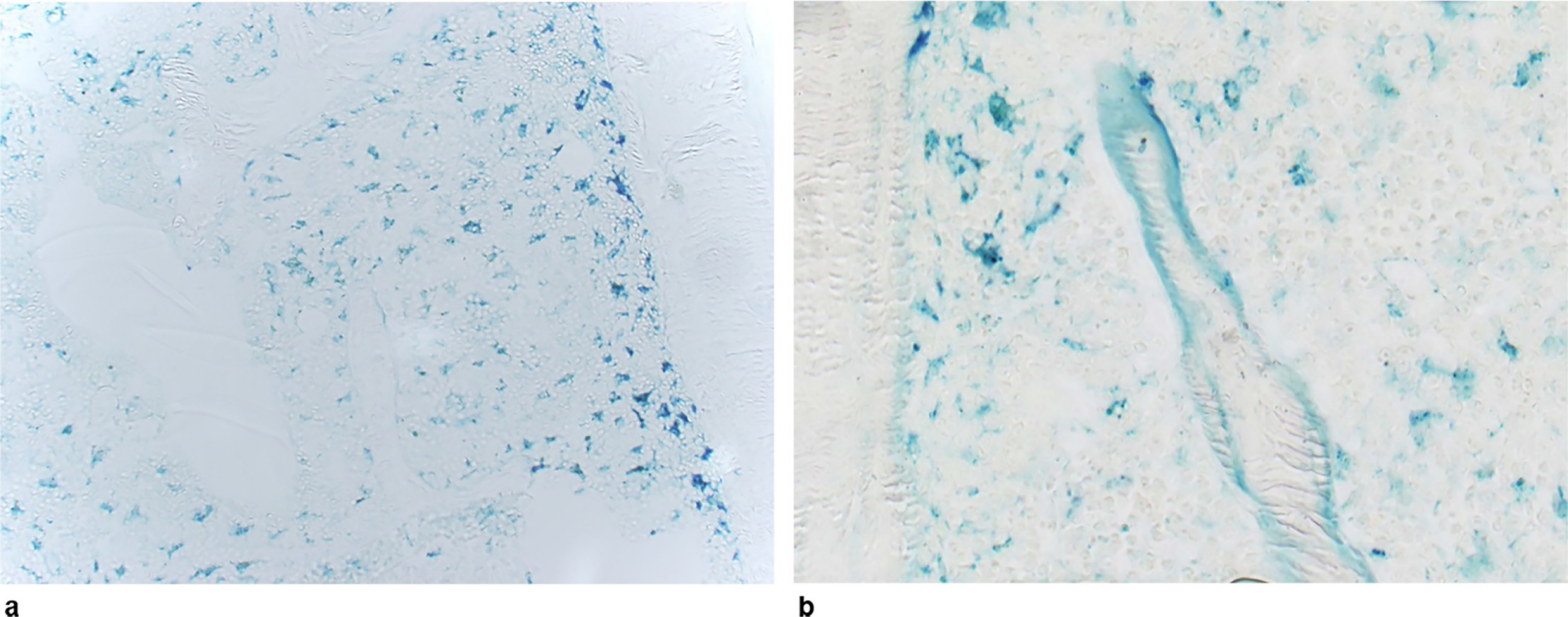
Histological detection of iron in bone using Perls’ staining on undecalcified sections. **(a)** Control mouse, the trabecular bone is unstained. **(b)** *Hfe*^-/-^ mouse, the trabecular surface is heavily labelled in blue, indicating considerable iron deposition. The blue cells in the marrow spaces are siderophages. Original magnification x200.

By studying Perl’s staining in control mice, we found no significant iron deposit on trabecular surfaces (Fig 1A). In contrast, in *Hfe*^-/-^ mice, the bone matrix at the trabecular surface was heavily labelled in blue, indicating a considerable iron overload (Fig 1B). This observation was confirmed by a significant difference in Fe.LS/BS between control and *Hfe*^*-/-*^ (Table 1).

### Bone histomorphometry

The results concerning microarchitecture are summarized in Table 2. In *Hfe*^*-/-*^ mice, we found a lower BV/TV in *Hfe*^*-/-*^ mice compared to controls. A significant increase in Tb.Sp was measured. Tb.N and Tb.Th were slightly decreased in *Hfe*^*-/-*^ mice compared to controls, but the difference did not reached significance. Significant disorganization of the trabecular network with a strongly lower ICI was found in *Hfe*^-/-^ mice compared to controls, and a reduced V*_m.space_. Using fractal parameters, D_M_ was significantly lower in *Hfe*^*-/-*^ mice indicating a reduced complexity of the trabecular network.

**Table 2:** Histomorphometric and histodynamic parameters in 6 months old control (C6M) and *Hfe*^*-/-*^ (H6M) male mice.

Mean ± SD	C6M (n = 7)	H6M (n = 7)	*p*
**BV/TV %**	***9*.*82 ± 1*.*56***	***7*.*86 ± 1*.*84***	***0*.*05***
**Tb.N 1/mm**	3.24 ± 0.32	2.82 ± 0.45	NS
**Tb.Th μm**	30 ± 4	28 ± 3	NS
**Tb.Sp μm**	***280 ± 31***	***332 ± 52***	***0*.*04***
**ICI**	***180*.*26 ± 48*.*75***	***397*.*90 ± 224*.*81***	***0*.*04***
V*_m.space_ **mm**^**3**^	***0*.*91 ± 0*.*19***	***1*.*35 ± 0*.*41***	***0*.*02***
V*_trab_ **mm**^**3**^	0.0058 ± 0.0023	0.00474 ± 0.002322	NS
**D**_**M**_	***1*.*27 ± 0*.*05***	***1*.*18 ± 0*.*08***	***0*.*02***
**OV/BV %**	3.69 ± 2.88	2.32 ± 2.75	NS
**O.Th μm**	4	4	NS
**Cn.MAR μm/day**	0.59 ± 0.08	0.61 ± 0.13	NS
**Ct.MAR μm/day**	0.67 ± 0.06	0.65 ± 0.30	NS
**dLS/BS %**	***22*.*00 ± 4*.*68***	***10*.*44 ± 6*.*14***	***0*.*002***
**BFR/BS mm**^**3**^**/mm**^**2**^**/day**	***48*.*26 ± 14*.*84***	***24*.*53 ± 15*.*36***	***0*.*01***
**N.Oc/B.Ar ȼ/mm**^**2**^	540.92 ± 165.26	666.69 ± 155.36	NS

**BV/TV:** Trabecular Bone Volume/Total Volume, **Tb.N:** Tabecular Number, **Tb.Th:** Trabecular Thickness, **Tb.Sp:** Trabecular Separation, **ICI:** InterConnectivity Index, V*_m.space_ and V*_trab,_: Star Volume of Marrow Spaces and Trabeculae_,_ **D**_**M**_: Minkowski’s fractal dimension, **OV/BV:** Osteoid Volume/Bone Volume, **O.Th**: Osteoid Thickness, **Cn.MAR** and **Ct.MAR:** Cancellous and Cortical Mineralization Rates, **dLs/BS:** Double Labelled Surface/Bone Surface, **BFR/BS:** Bone Formation Rate/Bone Surface, **N.Oc/B.Ar:** Number of Osteoclast/Bone Area

**Fig 2:**
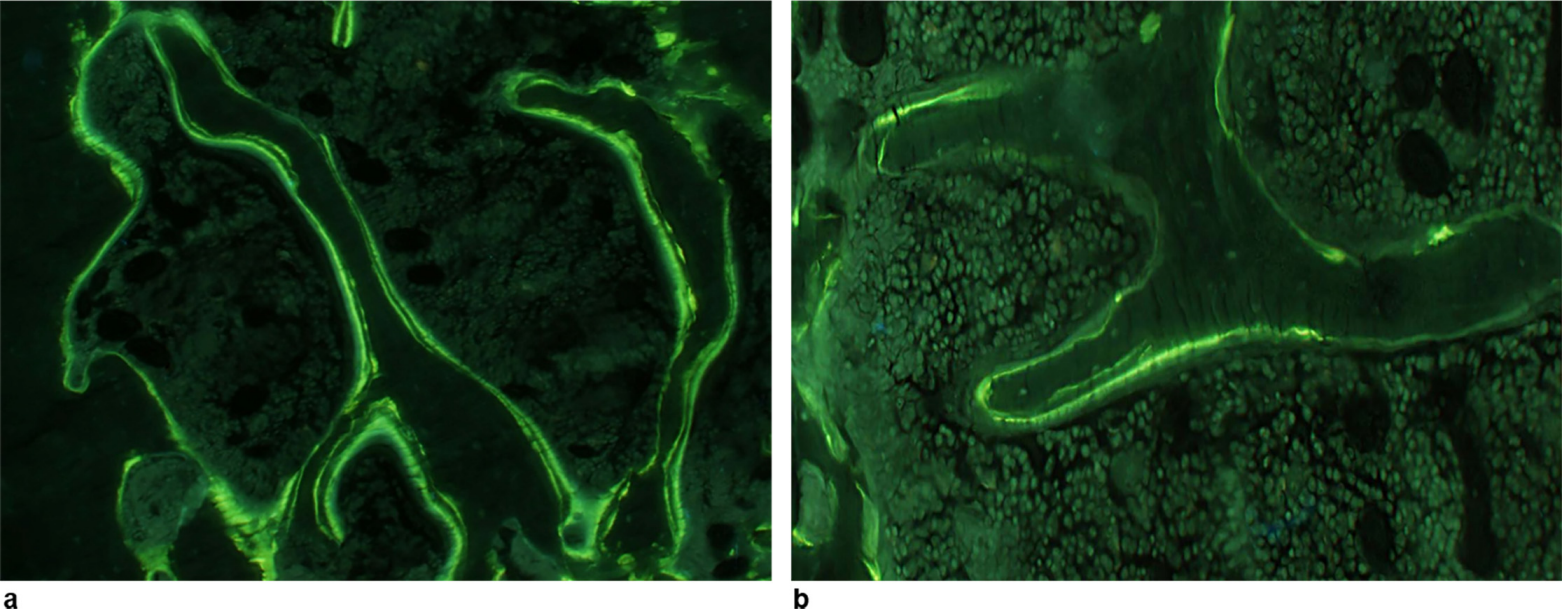
Histological detection of the mineralization front (green line) by calcein double labelling. **(a)** Control mouse. **(b)** *Hfe*^*-/-*^ mouse. Original magnification ×400.

**Fig 3:**
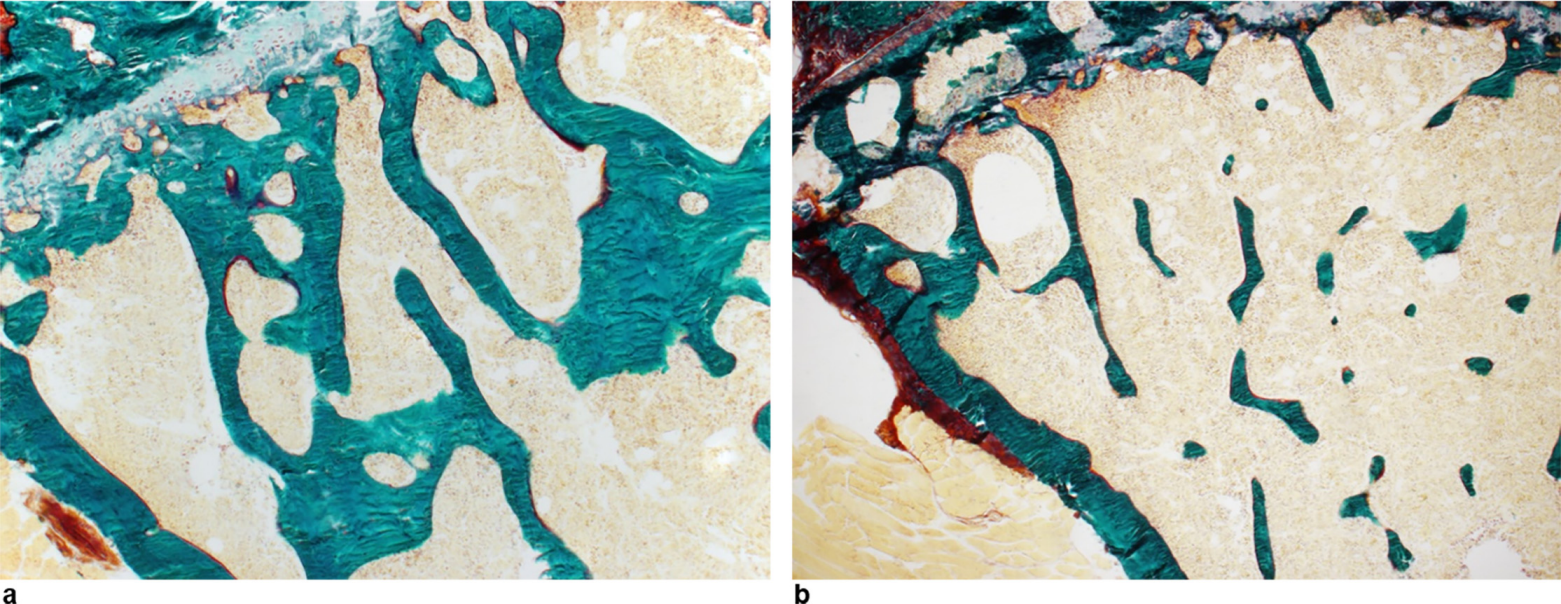
Histological detection of the osteoid section in bone using Goldner’s trichrome staining on undecalcified sections. **(a)** Control mouse. **(b)** *Hfe*^*-/-*^ mouse. Original magnification x100.

The main important findings were obtained on sections processed for double-labelled calcein (Fig 2). The MARs were normal, indicating that no mineralization defect occurred in *Hfe*^*-/-*^ mice. We found no differences in osteoid parameters, OV/BV and O.Th, between controls and *Hfe*^*-/-*^ mice (Table 2) (Fig 3). However, a very sharp decline the amount of double labelled surfaces indicated a net reduction in the number of active osteoblasts elaborating new bone structure units. The BFR/BS parameter, which is derived from MAR and dLS/BS, confirmed that the osteoblast defect in *Hfe*^*-/-*^ mice consists in a net reduction in the amount of bone formed by groups of osteoblasts acting at the surface of bone trabeculae. These cells elaborate less osteoid and mineralize it normally. The number of osteoclasts, identified histochemically, did not differ between the two groups but there was a non-significant tendency for osteoclastogenesis stimulation in *Hfe*^*-/-*^ mice.

## Discussion

Despite the fact that osteoporosis is reported to be a functional consequence of genetic or secondary hemochromatosis, the mechanisms involved are not fully understood. We aimed to analyse the impact of excess iron in *Hfe*^*-/-*^ mice on bone microarchitecture and osteoblast activity. Therefore, we studied the bone formation rate in *Hfe*^*-/-*^ mice. We found that bone contains excess iron associated with increased HIC, confirming that *Hfe*^*-/-*^ mice mimic human HG and represent a valuable model for studying relationships between osteoporosis and iron excess. This confirms our previous work on this model [26]. However the bone iron content is not uniform in this model and two of our mice had a poor iron overload.

Our results for bone mass parameters showed that iron deposition was associated with bone loss by producing microarchitectural impairment with a decreased BV/TV and Tb.N. A disorganization of trabecular network was found with an increase in the size of marrow cavities, which was confirmed by enhanced Tb.Sp and V*_m.space_. These microarchitectural changes led to a loss of connectivity and complexity in the trabecular network, which was confirmed by decreased ICI and increased Minkowski’s fractal dimension.

We have shown for the first time in a mouse model of GH that animals with iron overload have a decreased bone formation as evidenced by the decrease in double labelled surfaces and BFR/BS. This result was also observed in de Vernejoul et al.’s study [23] using an exogenous, non-genetic, iron-overloaded pig model. The model had a markedly decreased osteoblast surface and decreased mean wall thickness, a measurement of the quantity of bone deposited during one remodelling cycle. However, BV/TV was unchanged in this study. Perls deposits in the bone were not quantified. No mineralization impairment was evidenced in *Hfe*^*-/-*^ mice, in which OV/BV and O.Th remained unchanged. In Matsushima et al.’s study, male rats with exogenous iron overload had reduced BV/TV associated with increased bone remodelling, but not accompanied by any mineralization effect [24]. Later, in adults Wistar male rats, a loss of connectivity of trabecular bone at the femur associated with a decreased bone mineral density was observed in colloidal iron overloaded rats [25]. The differences in animal model and the nature of iron used to provoke experimental iron overload, and/or the judgment criteria could explain some differences in the results. One of the most important could be the genetic cause or not for iron overload.

We confirmed a negative direct impact of *Hfe*-related iron overload on bone mass and microarchitecture. Above all, we showed for the first time the negative impact of iron overload on bone formation in an animal genetic model known to mimic human GH. This finding strongly supports the hypothesis of osteoblast number/function impairment in osteoporosis related to iron overload during GH. Such *in vivo* finding are in accordance with and emphasize recent *in vitro* studies suggesting that excess iron has a direct impact on osteoblast function and, therefore, could decrease bone formation [18–21]. Studies on rat calvaria cultures and murine osteoblast cell lines have shown that iron exposure damages osteoblast cell viability, differentiation, and function by modulating gene expression [18–20]. Recently, we found similar results in a human osteoblast cell line exposed to excess iron and observed a decrease in the expression of genes involved in bone matrix formation or reported to be associated with osteoblast differentiation, such as collagen type I, osteocalcin, and RUNX2. In addition, we found that the expression of *HHIPL-2* (HedgeHog Interacting Protein Like-2) was modulated by iron overload; these results suggested that HHIPL-2 plays a role in decreased osteoblast function in bone formation [21]. The HHIPL2 exact biological function is not known. However, another member of the HHIPs family, HHIP, is known to limit the Hedgehog signalling pathway by interacting with the Hedgehog proteins [30]. This association could possibly also offer an explanation for HHIPL2’s role. This finding was also in agreement with studies on human mesenchymal stem cells that reported a down-regulation of Hedgehog signalling during osteoblast differentiation [31] and decreased expression of collagen type I and osteocalcin genes, which encode two bone matrix proteins, after the activation of Hh signalling [32].

These results fit in well with data reported in patients with thalassemia (the most common disease of secondary iron overload). Osteoporosis is frequent in such situations (40–50%) and causes high morbidity in children and adults [33,34]. Low bone mineral density [35,36], high fracture prevalence [37,38], and changes in the microarchitecture have been observed in these patients. A bone histomorphometry study in children and adolescents with β-thalassemia reported evidence of impaired osteoblast activity. A decrease in the BFR was found, but also defective mineralization associated with iron deposition on the mineralization front [13]. Despite the fact that other factors, such as hypogonadism, hyperparathyroidism, vitamin D deficiency, delayed puberty, defective growth hormone axis, and insulin growth factor 1 deficiency may favour osteoporosis in thalassemia [39], excess iron could be an independent factor.

Notably, an increase in iron-related osteoclast activity could also participate in the development of osteoporosis. Studies have reported that iron-related bone loss and trabecular microarchitectural changes are associated with increased resorption. In a mouse model of exogenous iron overload, bone alterations involving osteoclasts were found to be related to oxidative stress [40]. An increase in the RANKL/OPG ratio was found in thalassemia patients, suggesting an induction of osteoclast resorption activity [41–43]. In male *Hfe*^*-/-*^ mice, the osteoporotic phenotype with low bone mass and microarchitectural alterations was associated with increased osteoclast number [26]. In the present study, we found a trend of increased osteoclast number in *Hfe*^*-/-*^ mice compared to controls, but it was not significant. The low number of animals, due to a poor bone iron content for two *Hfe*^*-/-*^ mice, could explain this lack of significance.

In conclusion, for the first time in a genetic hemochromatosis mouse model, our results suggest that iron overload predominantly decreases bone formation, with alterations in bone mass and microarchitecture. These observations support a negative effect of iron on osteoblast recruitment and/or function, which may contribute to iron-related osteoporosis.
